# Manganese‐Enhanced Magnetic Resonance Imaging of the Heart

**DOI:** 10.1002/jmri.28499

**Published:** 2022-10-31

**Authors:** Trisha Singh, Shruti Joshi, Lucy E Kershaw, Marc R Dweck, Scott I Semple, David E Newby

**Affiliations:** ^1^ BHF/University Centre for Cardiovascular Science University of Edinburgh UK; ^2^ Edinburgh Heart Centre Royal Infirmary of Edinburgh UK; ^3^ Edinburgh Imaging University of Edinburgh UK

**Keywords:** manganese‐enhanced magnetic resonance imaging, calcium handling, kinetic modeling

## Abstract

Manganese‐based contrast media were the first in vivo paramagnetic agents to be used in magnetic resonance imaging (MRI). The uniqueness of manganese lies in its biological function as a calcium channel analog, thus behaving as an intracellular contrast agent. Manganese ions are taken up by voltage‐gated calcium channels in viable tissues, such as the liver, pancreas, kidneys, and heart, in response to active calcium‐dependent cellular processes. Manganese‐enhanced magnetic resonance imaging (MEMRI) has therefore been used as a surrogate marker for cellular calcium handling and interest in its potential clinical applications has recently re‐emerged, especially in relation to assessing cellular viability and myocardial function. Calcium homeostasis is central to myocardial contraction and dysfunction of myocardial calcium handling is present in various cardiac pathologies. Recent studies have demonstrated that MEMRI can detect the presence of abnormal myocardial calcium handling in patients with myocardial infarction, providing clear demarcation between the infarcted and viable myocardium. Furthermore, it can provide more subtle assessments of abnormal myocardial calcium handling in patients with cardiomyopathies and being excluded from areas of nonviable cardiomyocytes and severe fibrosis. As such, MEMRI offers exciting potential to improve cardiac diagnoses and provide a noninvasive measure of myocardial function and contractility. This could be an invaluable tool for the assessment of both ischemic and nonischemic cardiomyopathies as well as providing a measure of functional myocardial recovery, an accurate prediction of disease progression and a method of monitoring treatment response.

**Evidence Level: 5:**

**Technical Efficacy: Stage 5:**

Cardiac magnetic resonance imaging (MRI) has a major role in the diagnosis, evaluation, and tissue characterization of a range of cardiovascular diseases.^1^ Conventional cardiac magnetic resonance with gadolinium‐based contrast media can be complemented by T1‐mapping techniques, which allow the quantitative assessment of myocardial tissue. Furthermore, T1 mapping can risk stratify and provide prognostic value in conditions, such as dilated cardiomyopathy or hypertrophic cardiomyopathy.^1^

Manganese, a calcium analog, has paramagnetic properties and can cross intact cell membranes via calcium channels, providing intracellular contrast of viable tissue on MRI.^2^, ^3^ Manganese was introduced as liver‐specific contrast agent to visualize hepatic tumors and has subsequently been applied to preclinical neuronal assessment,^4^ and pancreatic beta‐cell activation.^5^ The major interest in manganese‐enhanced magnetic resonance imaging (MEMRI) of the heart lies in its biological function. In 1981, Hunter et al^6^ proposed that uptake of free manganese ions in the heart can be used to measure calcium uptake because manganese is retained intracellularly, and myocytes can be labeled without reduction in cardiac function by maintaining a very low manganese concentration in the perfusate.

This review provides a synopsis of previously published work and discusses the potential future clinical applications of MEMRI of the heart.

## Historical Background

Manganese was the first element used as a contrast agent in MRI.^2^, ^3^, ^7^ Certain characteristics make it a desirable contrast agent. Like gadolinium, it has paramagnetic properties thereby shortening T1 relaxation of water, enabling increased contrast in tissues where it accumulates and enhancing anatomical delineation.^2^ Manganese is also a naturally occurring trace element in the human body, which is required for several biochemical processes through its role as a co‐factor in the activation of several classes of enzymes.^8^ Humans obtain most of their manganese requirements through the diet and it is eliminated predominantly through the kidneys and liver.^9^ These properties contrast with gadolinium, which is toxic in its free unbound form and has no known biological function in humans.

**FIGURE 1:**
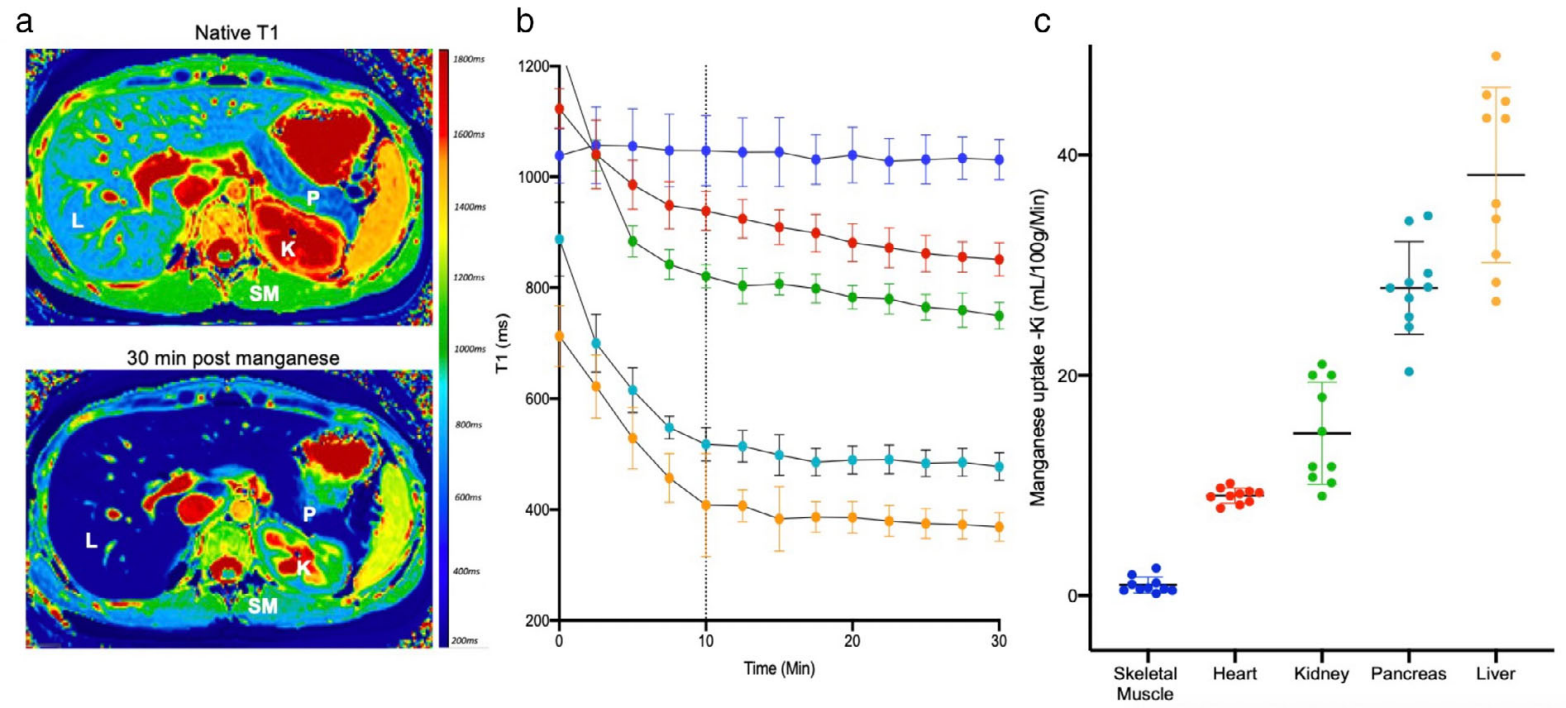
Manganese uptake in the body. Manganese‐enhanced images (a) at native T1 and post‐manganese T1 (30 minutes) demonstrating manganese enhancement in the liver (L), pancreas (P) and kidneys (K). Conversely, little enhancement is seen in skeletal muscle (SM). T1 decay curves (b) demonstrate greatest reduction in T1 in liver (green), followed by pancreas (light blue), kidney (green), heart (red) and skeletal muscle (dark blue). Dotted line represents end of manganese dipyridoxyl diphosphate infusion. Manganese uptake (c) in liver (green), pancreas (light blue), kidney (green), heart (red) and skeletal muscle (dark blue).

An important imaging characteristic of manganese ions is that they behave in a similar manner to calcium ions and are actively taken up by L‐type voltage‐gated calcium channels and sodium–calcium exchangers.^10^, ^11^ This means that uptake is seen in viable tissues, particularly those with a predominance of calcium channel activity, such as the liver, pancreas, brain, kidney, and heart (Fig. 1). Although also an analog of calcium, gadolinium ions are fully chelated in gadolinium‐based contrast media, because of the need to prevent gadolinium ion dissociation and any associated toxicity. As such, gadolinium‐based contrast media do not cross the cell membrane and accumulate in the extravascular extracellular space before returning to the circulation for excretion by the kidneys. Due to its strong paramagnetic properties and subsequent commercial development, gadolinium‐based media became the preferred contrast agent, eventually leading to a decline in interest for manganese‐based contrast media.

## Formulations

The formulations of manganese‐based contrast media define how they behave in vivo. In particular, they determine whether there is any associated toxicity, whether they can act as an intracellular contrast agent or whether they behave as an extracellular contrast agent.

### Nonchelated Forms of Manganese

Manganese chloride was one of the earliest manganese‐based contrast media to be used in MRI and can be administered orally. Once absorbed, manganese chloride freely dissociates into manganese and chloride ions. The manganese ions are strongly paramagnetic and actively enter myocytes via voltage‐gated calcium channels. However, due to competition with calcium, early studies demonstrated acute cardiac compromise, especially with high doses of manganese.^12^ As such, MEMRI remained underdeveloped for many years due to initial safety concerns and concomitant advances in gadolinium‐based imaging techniques.

### Partially Chelated

**FIGURE 2:**
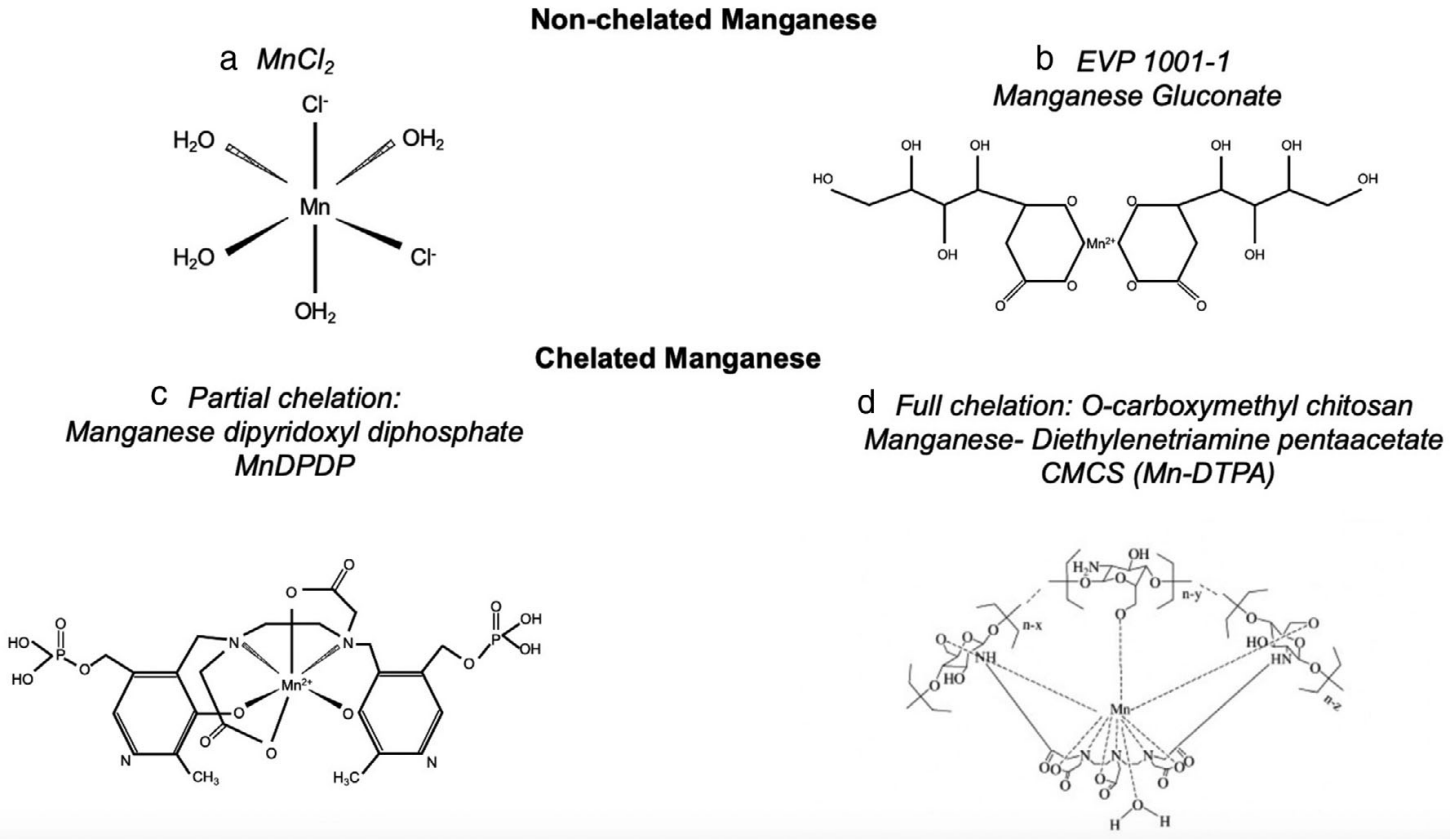
Types of manganese‐based contrast media. Manganese chloride (a), manganese gluconate (b), manganese dipyridoxyl diphosphate (c) and *O*‐carboxymethyl chitosan manganese‐diethylenetriamine pentaacetate are examples of nonchelated and chelated manganese‐based contrast agents, respectively. MnCl_2_ = manganese chloride; EVP 1001‐1 = manganese gluconate; MnDPDP = manganese dipyridoxyl diphosphate; CMCS (Mn‐DTPA) = *O*‐carboxymethyl chitosan manganese‐diethylenetriamine pentaacetate.

Subsequent work has sought to overcome the potential acute toxicity of high‐dose manganese ions. For intracellular myocardial imaging, manganese must be freely available for cardiomyocyte uptake. To date, two different methods have been employed producing distinct clinical‐grade agents, both with excellent safety profiles: 1) co‐administration with calcium and 2) partial chelation (Fig. 2, Table 1).

**TABLE 1:** Manganese‐Based Contrast Agents

Contrast Agent	Clinical Dose (μmol/kg)	Approved for Clinical Use	Previous or Currently Recruiting Studies
Chelated			
Partial: MnDPDP	5	Yes	Manganese‐enhanced MRI (MEMRI) of the myocardium (NCT03607669)
			MEMRI in ischemic, inflammatory and Takotsubo Cardiomyopathy (NCT04623788)
			The DAPA‐MEMRI Trial (NCT04591639)
			PANCREAS MEMRI (NCT05298735)
Full: CMCS (Mn‐DTPA)	0.03	No	None
Nonchelated			
MnCl2†	5	No	None
EVP1001‐1	1–10	No	Clinical Trial of MEMRI to assess peri‐infarct Injury (NCT02933034) Efficacy of EVP1001‐1 in the Assessment of myocardial viability in patients with cardiovascular disease (NCT01989195)

MnDPDP = manganese dipyridoxyl diphosphate; CMCS(Mn‐DTPA) = *O*‐carboxymethyl chitosan manganese‐diethylenetriamine pentaacetate; MnCl2† = manganese chloride.

#### CO‐ADMINISTRATION WITH CALCIUM GLUCONATE

To counter the adverse effects of free manganese ions, intravenous manganese co‐administered with calcium gluconate can result in a short plasma half‐life and rapid myocardial uptake with little redistribution.^13^ This has been developed for clinical use as EVP 1001‐1 (SeeMore, Eagle Vision Pharmaceuticals, Downingtown, USA), and it has reduced the toxic effects of high‐dose manganese ions and achieved sufficient T1 relaxation for imaging. However, not much work has been done with this formulation.

#### MANGANESE DIPYRIDOXYL DIPHOSPHATE

Unlike stronger chelates, which are designed not to dissociate, dipyridoxyl diphosphate chelation allows manganese to uncouple and circulate as a protein‐bound complex.^14^ The dynamics of myocardial uptake of manganese dipyridoxyl diphosphate have been described in detail elsewhere.^11^ In brief, after intravenous administration in humans, manganese dipyridoxyl diphosphate undergoes dephosphorylation and transmetallation with zinc to release manganese ions into the plasma.^14^, ^15^, ^16^, ^17^, ^18^ This controlled release of manganese ions facilitates rapid intracellular manganese uptake and renal clearance.^15^, ^16^ Administering manganese in this way successfully mitigates toxicity while retaining crucial paramagnetic and biological properties for adequate intracellular contrast.

Extensive research has been done in assessing dose concentration effects of manganese on longitudinal relaxation. Early preclinical studies demonstrated a close correlation between tissue manganese content and intracellular longitudinal relaxation (R1).^3^, ^16^, ^19^ Furthermore, they reported close to a linear correlation between intracellular R1 (1/T1) and ex vivo manganese concentration.^20^, ^21^ Studies in man have shown significant myocardial shortening of T1 relaxation at 5 μmol per kg bodyweight of manganese dipyridoxyl diphosphate (current approved dose), which is not linearly proportional to the total manganese infused. This contrasts with the marked dose–response seen in liver tissue.^22^ In humans, 70% of cytosolic calcium is from sarcoplasmic reticulum stores and approximately 30% is from extracellular uptake.^11^ This, and the higher distribution of calcium channel activity in the liver, pancreas and kidneys, could explain the relatively lower uptake of manganese in the myocardium (Fig. 1).

Manganese taken up by heart cells is retained for hours and does not redistribute, unlike calcium, which rapidly redistributes between intra and extracellular compartments.^16^ Myocardial manganese uptake from manganese dipyridoxyl diphosphate is slower than from manganese chloride^23^ and is cleared in 24 h without significant organ retention. This is likely due to slow release of manganese ions from dipyridoxyl diphosphate and elimination via hepatobiliary and urinary excretion. To date, manganese dipyridoxyl diphosphate (Teslascan; General Electric Healthcare) is the only manganese‐based contrast medium to have been approved for magnetic resonance imaging in humans, with a primary indication for hepatic tumor imaging.^24^

### Fully Chelated

Biocompatible macromolecular manganese‐based contrast media have been generated using *O*‐carboxymethyl chitosan (CMCS), diethylenetriamine pentaacetate (DTPA), and manganese (Mn). According to in vitro studies, CMCS‐(Mn‐DTPA) exhibits good cellular and blood biocompatibility at doses necessary for MRI. The relaxivity of CMCS‐(Mn‐DTPA) is approximately 3.5 and 5.5 times higher than that of *gadolinium‐DTPA* and manganese dipyridoxyl diphosphate, respectively.^25^ However, this is only when it is chelated to DTPA. Similar to gadolinium‐DTPA, aqueous CMCS‐(Mn‐DTPA) is stable enough to prevent the release of any manganese ions. Thus, partial chelation of manganese enables it to act as an intracellular agent, whereas full chelation results in a stronger paramagnetic agent but one that images the extracellular rather than intracellular compartment and hence cannot estimate calcium influx.

## Safety

Historically, there have been safety concerns regarding the early forms of manganese‐based contrast media.^26^, ^27^ Brurok and Jyunge conducted a series of preclinical studies of the cardiotoxic effects of manganese both during and after exposure of perfused rodent hearts to different concentrations of manganese chloride and manganese dipyridoxyl diphosphate.^26^, ^27^, ^28^, ^29^ They found that manganese chloride was 8–10 times more potent and competed too strongly with myocardial calcium uptake, causing myocardial depression, hypotension, and cardiac arrest.^27^ Onset of cardiac depression was immediate upon introduction of 30 mM manganese into the perfusate and normal function was restored within minutes upon removal of manganese chloride.^27^

Subsequent preclinical studies utilizing partially chelated manganese dipyridoxyl diphosphate demonstrated that manganese‐induced negative inotropy can be avoided by maintaining low extracellular manganese concentrations, and over time, contrast enhancement of the myocardium rapidly grows to detectable levels.^30^ Partially chelated manganese‐based contrast agents have been used in several clinical studies in patients with varying cardiac pathologies and demonstrate an excellent safety profile.^12^ There have been no reports of cardiac toxicity or changes in heart rate, blood pressure or cardiac contractility during manganese dipyridoxyl diphosphate infusion despite administration to patients with acute myocardial infarction or those with poor left ventricular systolic dysfunction.^31^, ^32^, ^33^

Although gadolinium‐based contrast agents are safe and well tolerated, it is important to remember that early formulations were toxic. It should also be remembered that gadolinium has no known physiological function or regulated secretory pathways in humans. In its free state, gadolinium is highly toxic. There are several potential mechanisms for this,^34^, ^35^ and the most well recognized is gadolinium ions interfering with many calcium ion channel‐dependent processes.^36^ For nearly a decade, there was an association between gadolinium‐based contrast media and the development of nephrogenic systemic fibrosis in patients with severe renal impairment.^37^ This risk was much higher in patients given acyclic gadolinium‐based contrast media. As such, these formulations (Gd‐DTPA, Gd‐DTPA‐BMA, and Gd‐DTPA‐BMEA) have been suspended by the European Medicines Agency and cases of nephrogenic systemic fibrosis have subsequently fallen dramatically.

It was previously widely believed that gadolinium‐based contrast agents are rapidly and completely eliminated from the human body. However, gadolinium can accumulate in tissues, such as the brain, bone,^38^ and kidneys,^39^ in patients who have received gadolinium‐based contrast media despite normal renal function. Furthermore, retention of gadolinium can be higher in those who have repeated exposure.^40^ Further work is needed to clarify propensity of macrocyclic agents to accumulate in the central nervous system and to establish if this is associated with any clinical sequelae. These safety concerns highlight our current dependence on gadolinium and the relative lack of alternatives.

Most human exposure to manganese occurs naturally as it is present in nearly all diets, with stable levels maintained by homeostatic mechanisms almost regardless of intake.^41^ Manganese is an essential mineral required for a range of enzymes central to normal physiological function of the body. Naturally occurring deficiency is rare, but toxicity from overexposure following environmental or occupational contact is recognized and can result in manganese accumulation in the globus pallidus, manifesting as headaches, gait disturbance and extra‐pyramidal symptoms and is referred to as “manganism.”^42^ However, neurotoxicity due to inhalation exposure has only been observed in miners,^43^ smelters,^44^ and welders^45^ over the course of years. Furthermore, previous studies have demonstrated a significant correlation between airborne manganese levels and manganism in welders, with estimated exposure dosages of 5–20 kg (containing 0.3–6% Mn) per working day per person.^46^, ^47^

Risk of manganese accumulation with intravenous manganese‐based contrast agents is minimal.^48^ Two factors aid in preventing this. First, total manganese exposure is very low even with repeated contrast doses. Second, manganese is naturally eliminated from the body through several regulatory processes. Furthermore, phase III clinical trials evaluated safety in patients with renal and hepatic impairment and demonstrated no clinical or laboratory evidence suggestive of manganese toxicity^48^ in any of the 48 patients with liver cirrhosis, including six subjects with hepatic failure. A more recent study has reported that an intravenous infusion of a standard imaging dose (5 μmol/kg) of manganese dipyridoxyl diphosphate in healthy human volunteers raised signal intensity in exocrine glands in the head and neck, in the choroid plexus, and in the anterior pituitary gland but not beyond the intact blood–brain barrier.^49^ However, there is a lack of long‐term safety data with the use of manganese‐based contrast agents and future work should focus on establishing this.

## Myocardial Calcium Handling

**FIGURE 3:**
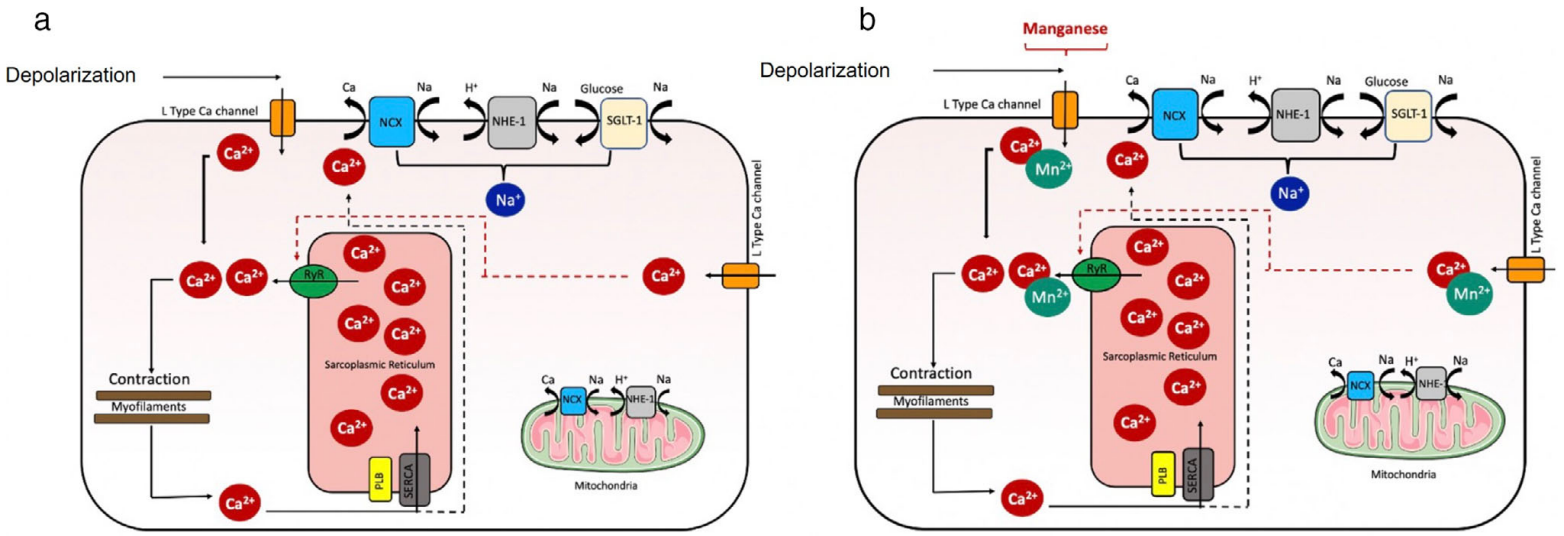
Myocardial calcium homeostasis and manganese uptake. Calcium homeostasis in normal myocardium (a) and manganese uptake via voltage‐gated calcium channels (b). Ca^2+^ = calcium ion; Na^+^ = sodium ion; H^+^ = hydrogen ion; Mn^2+^ = manganese ion; NCX = sodium–calcium exchangers; NHE‐1 = sodium hydrogen exchanger; RyR = ryanodine receptors; PLB = phospholamban; SERCA = sarcoplasmic reticulum calcium adenosine triphosphatase.

Myocardial contraction is controlled by excitation–contraction coupling which allows for rapid changes in calcium ion concentrations in the sarcoplasmic reticulum leading to contraction (systole) and relaxation (diastole, Fig. 3).^50^ Calcium homeostasis in the myocardium is essential for this and is controlled by several mechanisms. During systole, calcium ions are actively transported into the sarcoplasmic reticulum via L‐type voltage‐gated calcium channels. Calcium ions bind to ryanodine receptors resulting in the efflux of an even higher concentration of calcium ions from the sarcoplasmic reticulum into the cytosol.^50^ “Calcium induced, calcium release” in turn activates calcium‐sensitive contractile proteins (troponin C, troponin NC), which leads to myocardial contraction. During diastole, sarcoplasmic reticulum calcium adenosine triphosphatase (SERCA 2a) facilitates calcium entry back into the sarcoplasmic reticulum in addition to their exit into the extracellular space via the sodium–calcium exchanger and mitochondrial uptake.^50^ The net result is a reduced calcium concentration in the cytosol and myocardial relaxation. Phospholamban, a regulatory protein, promotes calcium efflux, resulting in reduced myocardial contraction. When phosphorylated, it causes disinhibition of SERCA 2a and subsequent increase in myocardial contraction.

## Myocardial Manganese Uptake

The major interest in manganese lies in its biological functionality. In 1970, Ochi^51^ first demonstrated that manganese ions were taken up by L‐type voltage‐gated calcium channels in cardiomyocytes.^51^ Uptake of manganese was 35‐fold slower in the resting heart compared to the beating heart. Moreover, addition of agents known to influence calcium uptake by myocytes led to similarly detected alteration in manganese uptake.^6^, ^52^ Early ex vivo animal studies showed that accumulated manganese led to markedly increased longitudinal relaxation rates in normal myocardium compared to infarcted regions.^53^, ^54^ Thus, abnormal myocardium would result in reduced manganese uptake. This led to the idea that manganese could be used as a surrogate marker of calcium handling (Fig. 3).

## Manganese T1 Mapping and Patlak Modeling

Manganese causes shortening of T1 relaxation time of water protons due to its paramagnetic properties. MEMRI is therefore best visualized and quantified using T1‐weighted imaging. However, T1 mapping allows for quantitative assessment of manganese uptake in cells, because of the linear relationship between the longitudinal relaxation rate R_1_ (1/T_1_) and manganese concentration.^55^, ^56^ Many animal studies have confirmed that manganese‐enhanced imaging enhances tissue contrast by manganese uptake and retention in viable cells, resulting in shortening of T1.^23^, ^29^, ^30^, ^54^, ^57^, ^58^

**FIGURE 4:**
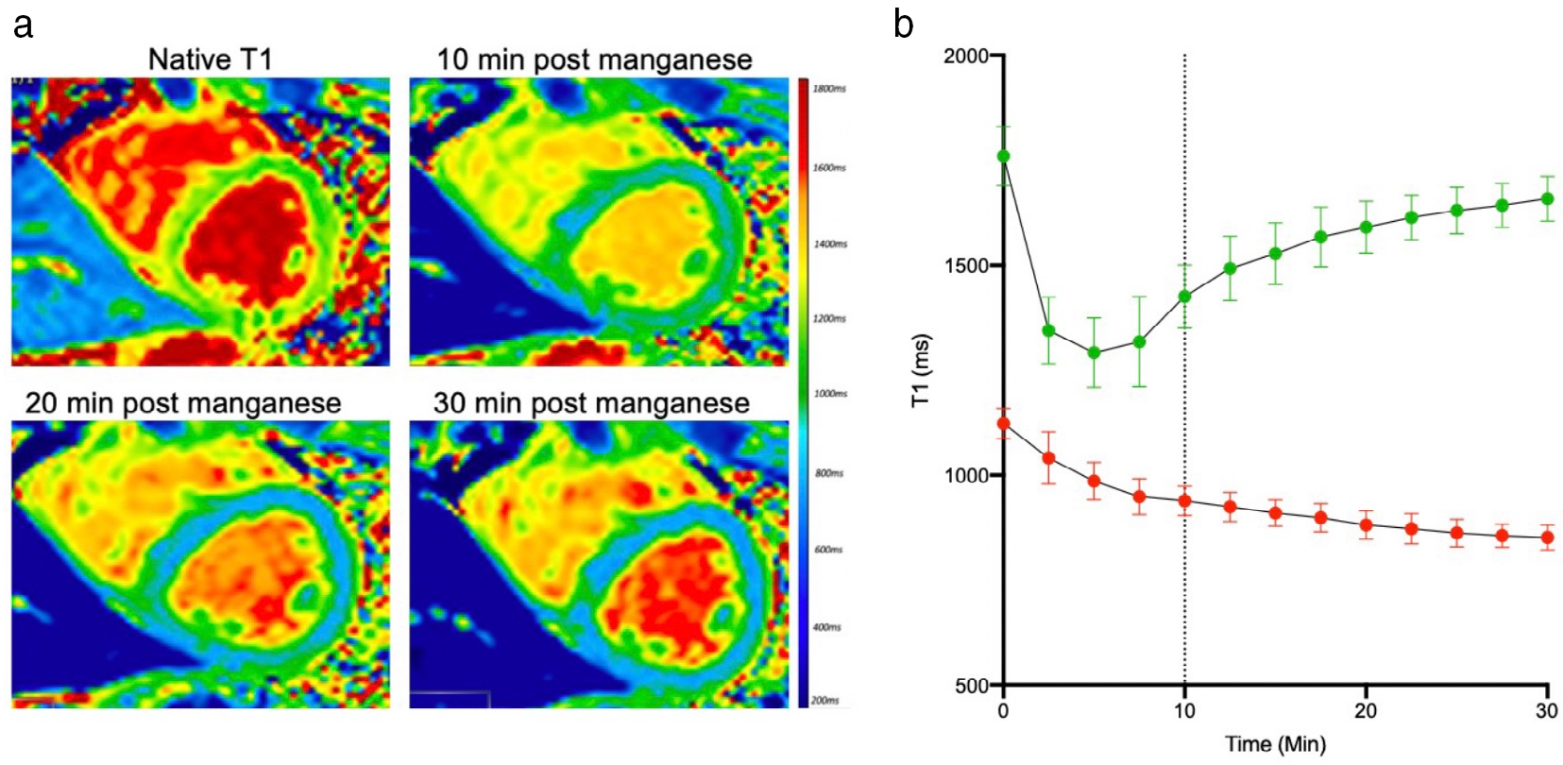
Manganese‐enhanced magnetic resonance imaging of healthy myocardium. Short‐axis T1 mapping in healthy myocardium with manganese dipyridoxyl diphosphate (a). Rapid reduction in T1 is seen in the blood pool (green, b), followed by rapid normalization by 30 minutes. In contrast, the T1 value of myocardium (red) shows steady and sustained reduction throughout the imaging time period (b). Dotted line represents end of manganese dipyridoxyl diphosphate infusion.

Skjold et al were the first to demonstrate the effectiveness of manganese in imaging the healthy human myocardium.^22^ They applied T1 mapping to short‐axis slices of the left ventricular myocardium in healthy volunteers before and after intravenous infusion of manganese dipyridoxyl diphosphate (5–15 μmol/kg).^22^, ^53^, ^54^ T1 values from bloodpool and myocardium measured over time demonstrated a sharp reduction in blood pool T1 during initial infusion phase followed by normalization to baseline by 30 minutes (Fig. 4). Myocardial T1 values also demonstrate a rapid initial descent (infusion phase), but this is followed by a plateau phase (34%–46%),^22^ likely attributable to ongoing intracellular myocardial uptake and accumulation.

**FIGURE 5:**
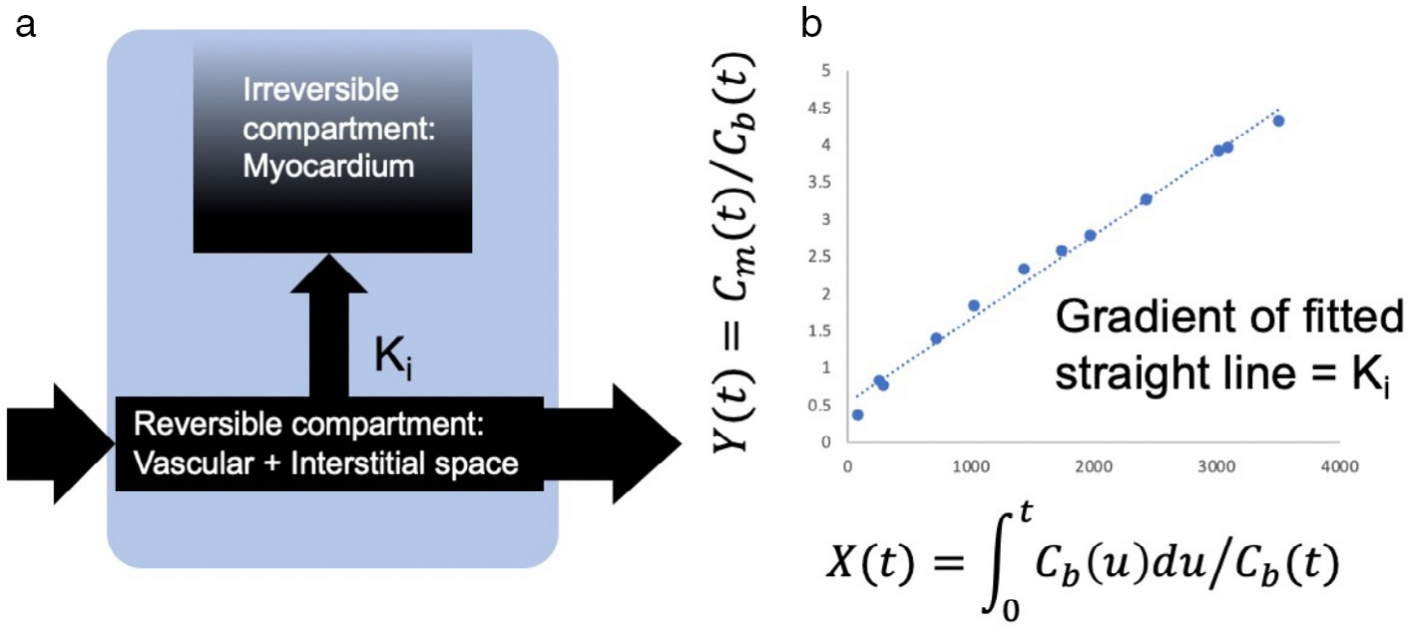
Patlak modeling. Patlak formulation—schematic of (a) model compartments and transfer constant *K*_*i*_, describing passage from reversible to irreversible compartment (b) data analysis. C_m_(t) = myocardial manganese concentration; C_b_(t) = blood manganese concentration.

Myocardial manganese uptake can be quantified by tracer kinetic modeling. Patlak et al were the first to provide a graphical analysis of unidirectionality of transfer and of the influx constant, using brain uptake data.^59^ The multitime approach produces information about the exchange rate of the compartments that rapidly and reversibly exchange with plasma. Furthermore, this can be used to assess the rate constant of any type of irreversible process within any organ system.^59^ The Patlak two‐compartment model has become the commonest modelling approach in cardiac manganese‐enhanced imaging. This assumes the influx of manganese ions from a reversible (*v*_e,_ extracellular and vascular space) into a largely irreversible compartment (*v*_i_, cardiomyocyte during the imaging period). This apparent unidirectional influx constant (*Ki*) for the transfer of manganese from plasma to irreversible compartments *v*_i_, can be measured, using Equation 1:

(1) $$\frac{C_{t}\left(t\right)}{C_{a}\left(t\right)}=\mathrm{Ki}\frac{\int_{0}^{t}C_{a}\left(\tau \right)\mathrm{d\tau}}{C_{a}\left(t\right)}+v_{e}$$

where *C*_*t*_ and *C*_*a*_ are the manganese concentration in myocardial tissue and blood pool (arterial input function), respectively. This formula is equivalent to the Patlak model^59^ and describes that if a contrast medium is irreversibly trapped in the tissue within the imaging period, the instantaneous tissue concentration (myocardial T1) divided by the instantaneous arterial concentration (bloodpool T1) plotted against the integrated arterial concentration divided by the instantaneous arterial concentration, will result in linearization of the data (Fig. 5).^54^, ^59^

Early animal studies applied a two‐compartment model to MEMRI of the retina and successfully estimated apparent retinal transfer constant *Ki*.^60^ Years later, Skjold was the first to apply tracer kinetic modelling to R1 curves, a unidirectional influx constant for manganese (*Ki*), was measured as an index of myocardial calcium channel activity in healthy volunteers.^54^ This demonstrated that the use of unidirectional manganese influx rates may be a valuable research tool for in vivo studies of myocyte functioning in myocardial disease.^31^, ^33^, ^54^ Furthermore, only minor differences in myocardial manganese uptake were seen between administration of manganese over 5 or 30 minutes.^31^, ^33^, ^54^

There are limited data on the repeatability and reproducibility of MEMRI, which is essential for its future clinical application. Spath et al performed interobserver reproducibility for healthy volunteers (*n* = 20) and patients with hypertrophic (*n* = 10) and dilated cardiomyopathy (*n* = 10). This demonstrated no significant difference in native T1 and post‐manganese T1 between two independent observers for healthy volunteers (*P* = 0.89 and 0.97, respectively) and patient cohorts.^33^ An ongoing sub‐study is evaluating intraobserver and interobserver reproductivity and scan–rescan repeatability of manganese‐enhanced magnetic T1 mapping and kinetic modeling in healthy volunteers and patients with acute myocardial infarction, hypertrophic and dilated cardiomyopathy (Singh T, unpublished data).

## Flow or Function

Manganese uptake in the heart is determined by both the amount of manganese being delivered to the myocardium (blood flow) and the amount that is taken up into the intercellular space (function), instead of passing through the circulation. This is crucial in interpreting patterns of manganese uptake according to differences in regional function and it is not a simple question since flow and function are highly interconnected, particular in patients with ischemic heart disease. The heart does not retain all the manganese that enters its circulation. Instead, the uptake of manganese is determined by competitive uptake by calcium channels, which can be approximated by a rate constant (influx from reversible to irreversible compartment).^54^, ^59^ However, manganese uptake (*Ki*) is an amalgamated measure that does not distinguish between flow and transfer into the cells.

The evidence for flow and function is sparse and contradictory. Preclinical studies reported relative low uptake of manganese observed in myocardium served by a partially restricted artery and this was likely to represent lower blood flow.^13^ Similarly, it would be reasonable to assume that reduced manganese uptake seen in patients with acute myocardial infarction can be ascribed to reduced flow due to coronary artery occlusion.^61^

As a calcium analog, the influx of manganese into the cardiomyocyte is through voltage‐gated calcium channels. As such, the rate of this influx could be altered under conditions that increase or decrease calcium influx into the heart. Calcium channel blockers are known to decrease cardiac function by decreasing calcium influx into the heart. Animal models have demonstrated that pretreatment of ex vivo mouse myocardium with diltiazem resulted in reduced manganese‐induced T1 shortening. This inhibition was similar between all agents with manganese chloride experiencing a maximal mean reduction of 30% in T1 shortening, while EVP1001‐1 and manganese dipyridoxyl diphosphate experienced reductions of up to 43% and 32%, respectively.^62^ Conversely, β‐adrenergic agonists, such as dobutamine, are known to increase calcium influx into the heart to increase contractile function. Hu et al^52^ demonstrated upregulation of myocardial manganese uptake with dobutamine, favoring flow‐related mechanism. Improved contrast between normal and ischemic myocardium with greater reduction in remote myocardial T1 during both dipyridamole and dobutamine stress has been reported with EVP1001‐1 in both acute and chronic models of ischemia.^13^ However, this has not been replicated in other studies. Further work is required to establish the true impact of flow vs. function.

## Cardiac Applications

With increasing interest in MEMRI over the last decade, this technique has been translated to assess myocardial calcium handling in early clinical studies.

### Myocardial Infarction

#### MYOCARDIAL CALCIUM HANDLING IN MYOCARDIAL INFARCTION

After a period of ischemia and hypoxia, myocardial cells within the infarct region demonstrate major metabolic abnormalities, resulting in myocardial calcium mishandling. Intracellular hydrogen ion aggregation causes a low intracellular pH, and intracellular sodium increases through sodium/hydrogen exchange.^63^ Excessive intracellular sodium will promote sodium excretion and calcium intake by sodium–calcium exchange, which increases intracellular calcium levels, leading to calcium overload. This can cause a series of irreversible cell injury responses, such as cardiac contractile dysfunction and apoptosis. Furthermore, when blood flow and oxygen supply to the cardiac tissue returns to normal intracellular calcium overload is further aggravated.^63^

The peri‐infarct zone is an area of heterogeneous myocardial scar containing fibrotic tissue intermingled with viable cardiomyocytes. This region is important because, in the acute phase, cells may demonstrate myocardial stunning. This refers to persistent myocardial contractile dysfunction transiently induced by acute ischemia despite reperfusion and absence of irreversible damage. It is clear that stunned myocardium is characterized by abnormalities in excitation–contraction coupling.^64^ However, despite intense research efforts, there remains some ongoing controversy as to the exact nature of these abnormalities. Contractile abnormalities can arise from either a decrease in calcium availability, such as from sarcoplasmic reticular dysfunction, or a decreased responsiveness of the myofilaments to calcium, such as from proteolysis of troponin I.^64^

#### ACUTE MYOCARDIAL INFARCTION

Animal models have assessed quantification of myocardial infarction with MEMRI.^61^, ^65^, ^66^ In a rat myocardial infarction model, manganese enhancement was present in the normal myocardium but absent in the infarcted tissue.^67^ In an ischemia reperfusion model, manganese dipyridoxyl diphosphate enhancement on in vivo and ex vivo MRI correlated well but was smaller than the areas on triphenyl tetrazolium chloride staining. This suggests that MEMRI detects both infarcted and stunned myocardium.^65^, ^66^ A recent murine study compared manganese‐enhanced and gadolinium‐enhanced T1 mapping measurements of myocardial infarction size at 1 hour, 1 day and 14 days after arterial occlusion. The authors reported that MEMRI provides an early biomarker on final infarct size after permanent coronary occlusion.^68^

**FIGURE 6:**
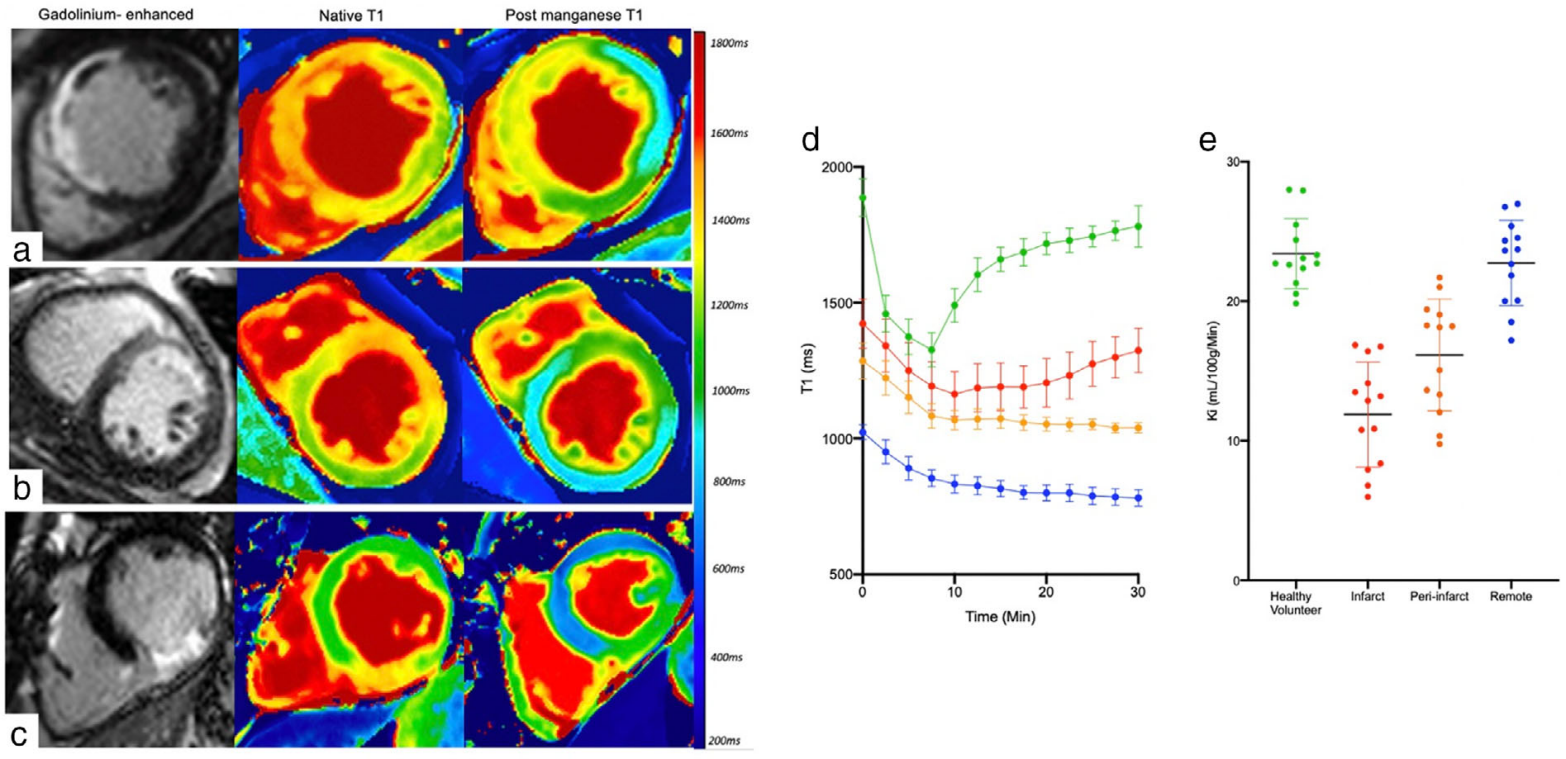
Manganese‐enhanced magnetic resonance imaging in acute myocardial Infarction. Short‐axis views of gadolinium‐enhanced, native and 30‐minute postmanganese T1 map images in patients with acute anteroseptal (a), anterior (b), and inferior (c) myocardial infarction. Gadolinium‐enhanced images demonstrate the presence of late gadolinium in the anteroseptal, anterior and inferior walls respectively. Conversely, manganese‐enhanced images demonstrate reduced manganese uptake (abnormal calcium handling, green) in the anteroseptal, anterior and inferior walls. Mean T1 decay times in bloodpool (green), infarct (red), peri‐infarct (orange) and remote region (blue) in patients with acute myocardial infarction (d). Mean myocardial manganese uptake (Ki‐ mL/min/100 g of tissue) defined by Patlak modeling in patients with acute myocardial infarction and healthy volunteers (e).

More recently, serial MEMRI has been used to investigate the time course of changes in patients with acute myocardial infarction.^32^ Regions with transmural infarction demonstrated partial recovery of T1 values similar to that of bloodpool (Fig. 6). The lack of manganese uptake here likely represents absence of myocardial calcium handling in the infarct area secondary to myocardial necrosis and absent myocyte viability. Unlike late‐gadolinium enhancement, where contrast agent accumulates in areas of myocardial necrosis, manganese uptake occurs in viable tissue, therefore absence or reduction of manganese uptake suggests absent or impaired calcium handling, thereby behaving as an inverse of gadolinium imaging (Fig. 6). Late‐gadolinium enhancement is the gold standard for visualization and quantification of myocardial infarction size.

Spath et al report that MEMRI was more sensitive than late‐gadolinium enhancement in detecting dysfunctional myocardium (dysfunctional fraction 40.5 ± 11.9 vs. infarct size 34.9 ± 13.9%; *P* = 0.02).^32^ This is likely due to the latter being nonspecific and overestimating infarct territory due to acute edema.^32^, ^62^ MEMRI tracked more closely with abnormal wall motion than late‐gadolinium imaging (mean *r* ^2^ = 0.72 vs. 0.55, *P* < 0.0001)^32^. A step‐wise reduction was seen in manganese uptake across remote, peri‐infarct and infarcted myocardium.^32^ After 3 months, myocardial manganese uptake in the peri‐infarct region was similar to that of the remote regions. This suggests that the lack of manganese uptake in the peri‐infarct area may represent myocardial stunning.

#### ESTABLISHED MYOCARDIAL INFARCTION

Beyond infarct quantification, manganese‐enhanced magnetic resonance has the potential to assess myocardial viability with potential application to myocardium with chronic myocardial contractile dysfunction secondary to ischemia: the so‐called “hibernating” myocardium. Such “hibernating” cardiomyocytes remain viable and often restoration of blood flow will lead to some degree of improvement in left ventricular ejection fraction. Thus, identifying myocardium with potential for improvement in contractility is vital to determine the appropriateness of coronary revascularization.

^18^F‐Fluorodeoxyglucose positron emission tomography (^18^F‐FDG PET) is the gold standard for assessing myocardial viability^69^ offering the greatest sensitivity for viable myocardium and comparable specificity to other imaging modalities. Although imaging in this way directly assesses viability through metabolic functionality of tissues (a mechanistically similar method of myocardial viability assessment to MEMRI), its use is often limited by availability and expertise. This, combined with the properties of manganese, led to the investigation of MEMRI to assess and quantify myocardial viability directly. Preclinical studies have validated direct quantification of myocardial viability using MEMRI compared with 18F‐FDG PET, suggesting both cellular calcium and glucose uptake are robust and concordant markers of myocardial viability.^62^

Due to radiation exposure and lack of ^18^F‐FDG PET availability, gadolinium became invaluable in identifying scarred nonviable myocardium. However, viability in gadolinium‐enhanced imaging is simply inferred and cannot be measured directly or quantitatively. Although data for chronic ischemia are limited, manganese uptake in the peri‐infarct region at 1 month does appear to be greater than infarct zone in animal models.^70^ This implies that MEMRI can enhance myocardium within injured but not infarcted myocardium and could be an important biomarker of viability.

Skjold et al^53^ were the first to describe MEMRI in patients with prior established myocardial infarction. They described the ability of manganese‐enhanced magnetic resonance to characterize and to quantify viable myocardium directly (Fig. 6). This confirmed the potential to detect viable myocardium, which could prove an invaluable tool in patient selection for revascularization therapies. Moreover, it holds particular promise as a biomarker of treatment efficacy for interventional strategies targeting ischemia‐reperfusion injury.

### Heart Failure

#### MYOCARDIAL CALCIUM HANDLING IN HEART FAILURE

Due to the fundamental role of calcium in excitation–contraction coupling, dysfunctional myocardial calcium handling is central to the pathophysiology of the failing myocardium. The amount of calcium entering the cytoplasm and its rate of removal are major factors in determining rate, intensity and duration of myocardial contraction.^71^

**FIGURE 7:**
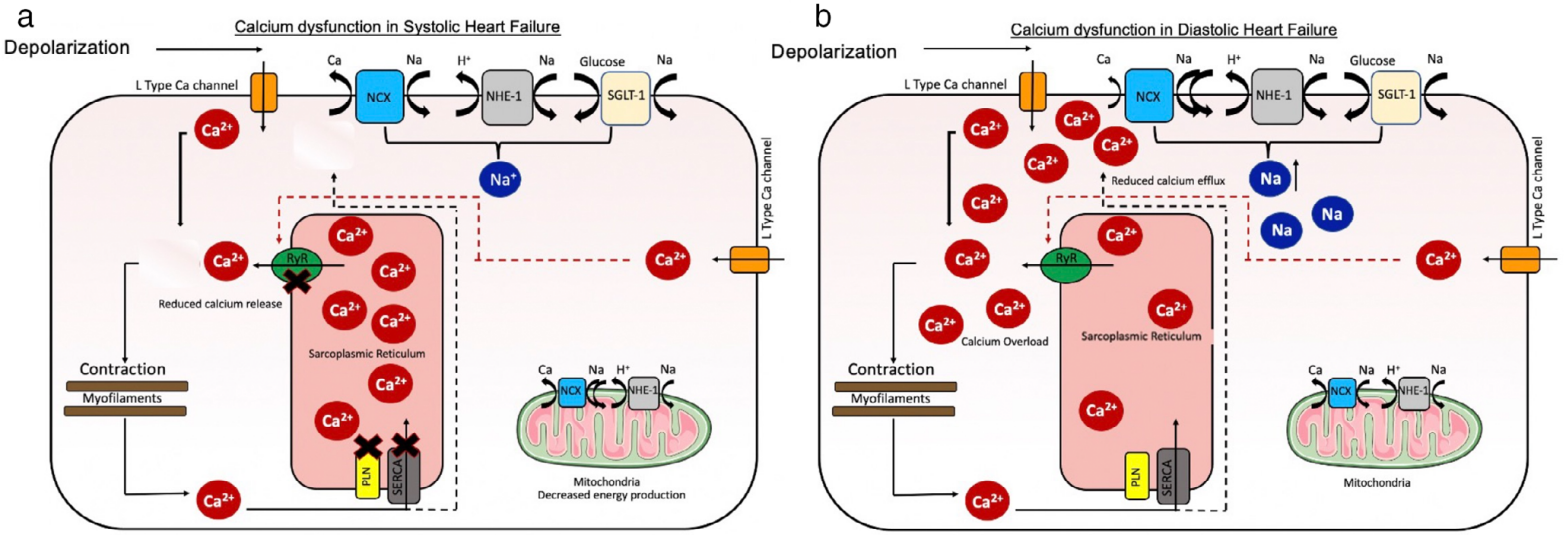
Calcium dysfunction in the failing myocardium. Several factors cause reduced calcium ion (Ca^2+^) release from the sarcoplasmic reticulum resulting in systolic heart failure (a). Diastolic heart failure results from reduced rate of Ca^2+^ removal causing delay in myocardial relaxation (b). Ca^2+^ = calcium ion; Na^+^ = sodium ion; H^+^ = hydrogen ion; Mn^2+^ = manganese ion; NCX = sodium–calcium exchangers; NHE‐1 = sodium hydrogen exchanger; RyR = ryanodine receptors; PLB = phospholamban; SERCA = sarcoplasmic reticulum calcium adenosine triphosphatase.

Several mechanisms contribute to disrupted calcium handling in systolic dysfunction. First, release of calcium from the sarcoplasmic reticulum is reduced which in turn impairs calcium‐induced calcium release.^72^ Second, phosphorylation of L‐type voltage‐gated calcium channels is increased during heart failure, which results in calcium leaking from the sarcoplasmic reticulum.^72^ Third, there is a substantial reduction in SERCA2a expression in patients with ischemic cardiomyopathy and to a lesser degree with dilated cardiomyopathy.^72^ On the other hand, phospholamban (PLN) expression and phosphorylation were relatively unchanged.^72^ This not only reduces intracellular uptake of calcium via SERCA2a but also increases PLN: SERCA ratio resulting in predominately calcium efflux and inhibition of myocardial contraction.^72^ Finally, although ryanodine receptor expression remains unchanged in the failing myocardium, its function is calcium dependent and therefore affected by intracellular calcium concentration. Together these factors result in reduced calcium release from the sarcoplasmic reticulum (Fig. 7).

Myocardial calcium handling is important in cardiac diastology. To achieve relaxation, cytosolic calcium must be sequestered, mainly to the sarcoplasmic reticulum by SERCA2.^73^ Diastolic calcium is increased in human heart failure, a condition that is likely related, at least in part, to defects in cytosolic calcium removal.^72^ Elevated intracellular sodium and altered sodium channel properties are present in failing myocardium of humans.^74^ Changes in intracellular sodium may have a large impact on calcium homeostasis.^74^ Small increases in sodium, increases calcium influx via reverse‐mode sodium–calcium exchange during systole and limits calcium extrusion via forward mode sodium–calcium exchange during diastole. The reduced rate of calcium removal reduces the rate of recovery and is associated with marked delay in myocardial relaxation. Calcium overload contributes to arrhythmias and diastolic dysfunction (Fig. 7).^75^

#### DILATED CARDIOMYOPATHY

Dilated cardiomyopathy is the commonest form of cardiomyopathy and is characterized by left ventricular dilatation and dysfunction in the absence of abnormal loading or ischemia. Dysfunctional calcium handling is a central feature of left ventricular dysfunction in patients with dilated cardiomyopathy, with alterations in calcium handling proteins leading to reduced myocardial contractile function.^76^ As such, the ability to detect and to monitor calcium handling noninvasively has great potential to be used as a marker for risk stratification, disease progression and response to therapy.

Gadolinium‐enhanced MRI is used to identify fibrosis and the degree of fibrosis is a predictor of all‐cause mortality. Similarly native T1 can detect diffuse subclinical fibrosis^77^ and is an important predictor of ventricular arrhythmias. There are no studies that assess MEMRI in animal models of dilated cardiomyopathy.

**FIGURE 8:**
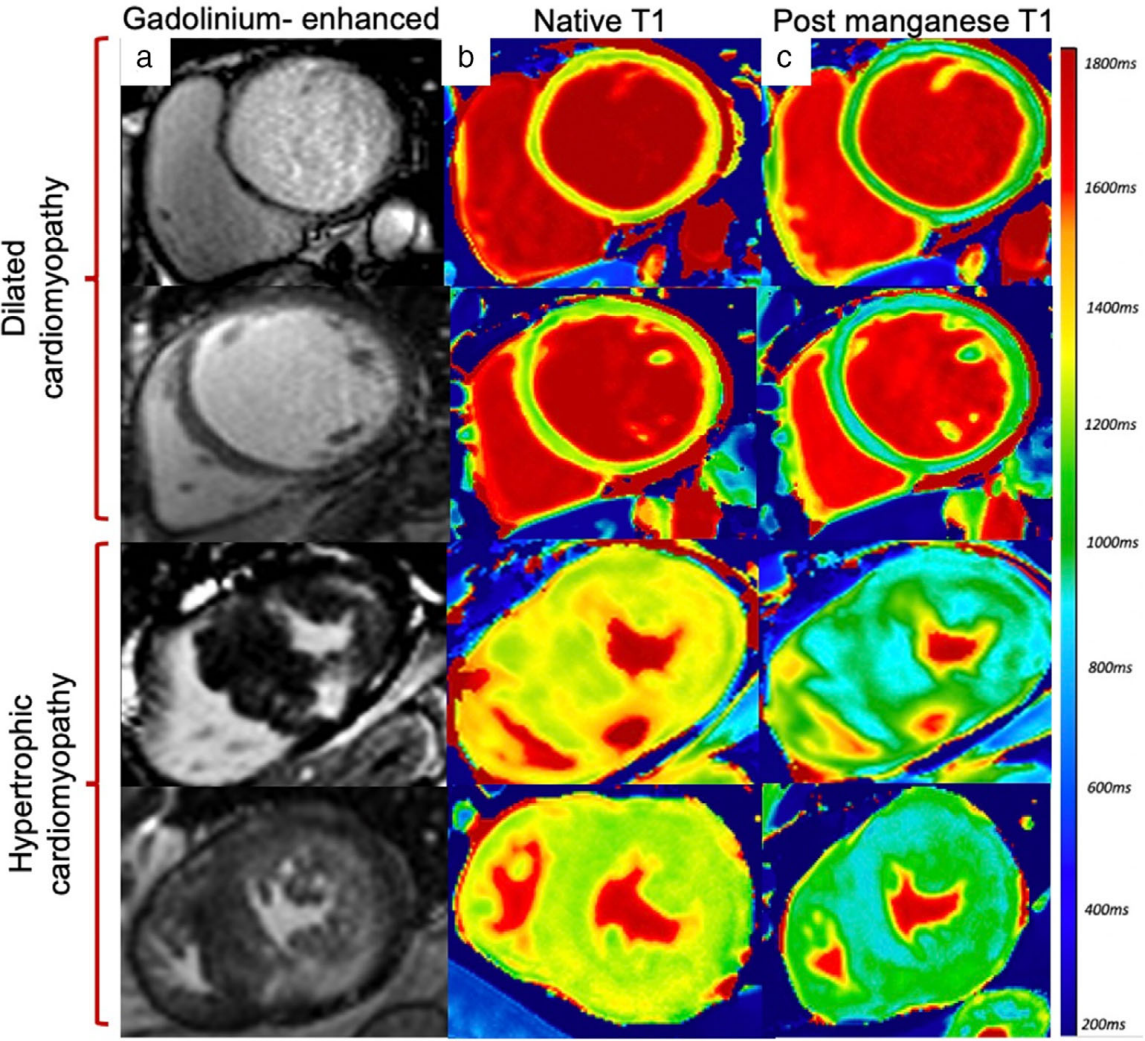
Manganese‐enhanced magnetic resonance imaging in ischemic and nonischemic Cardiomyopathy. Short‐axis views of gadolinium‐enhanced images (a), native T1 (b) and 30‐minute post‐manganese T1 maps (c) in patients with dilated and hypertrophic cardiomyopathy.

A small pilot study investigating MEMRI in patients with dilated cardiomyopathy^33^ demonstrated reduced myocardial manganese uptake, suggestive of dysfunctional myocardial calcium handling (Fig. 8). Furthermore, MEMRI correlated with left ventricular ejection fraction in patients with dilated cardiomyopathy, suggesting it has potential to track and to quantify more subtle gradations of myocardial dysfunction. The cellular mechanisms underlying this have yet to be explored in detail, but it points to important potential of MEMRI for noninvasive detection of calcium dysregulation. However, this is not specific to nonischemic cardiomyopathies and could potentially be used to assess a wider range of cardiomyopathy phenotypes, although this has yet to be established.

#### HYPERTROPHIC CARDIOMYOPATHY

Hypertrophic cardiomyopathy is a primary myocardial disorder characterized by myocyte remodeling, disorganization of sarcomeric proteins, impaired energy metabolism and altered cardiac contractility. Genetic mutations in patients with hypertrophic cardiomyopathy result in increased mitochondrial activity and altered myofilament calcium sensitivity involving both calcium‐dependent and independent processes,^78^ resulting in increased left ventricular wall thickness with associated diastolic and systolic dysfunction. Furthermore, dysfunctional calcium handling appears to precede alterations in metabolic activity and is associated with an increased risk of arrhythmogenesis.^78^

Myocardial fibrosis is directly linked to the increased risk of ventricular tachyarrhythmia and sudden cardiac death.^79^ Native T1 is often abnormal, indicating early fibrosis and has shown potential in adding to current risk stratification.^80^ There are no animal models of hypertrophic cardiomyopathy, but MEMRI has been evaluated in a murine model of myocardial hypertrophy. Pharmacological induction of hypertrophy was achieved using an infusion of isoproterenol. Following manganese dipyridoxyl diphosphate administration, there was clear reductions in myocardial T1 relaxivity and reduced manganese uptake in both the septum and the free‐wall of the left ventricle, mimicking hypertensive heart disease.^81^

To date there has only been one study assessing MEMRI in patients with hypertrophic cardiomyopathy. This demonstrated reduced myocardial manganese uptake in areas of hypertrophy, suggesting abnormal myocardial calcium handling (Fig. 8). All patients had normal left ventricular function, suggesting that abnormal myocardial calcium handling was predominantly due to diastolic impairment. Diastolic dysfunction in hypertrophic cardiomyopathy is multifactorial and includes prolonged and disordered ventricular relaxation, decreased chamber compliance and abnormal calcium cycling.

Interestingly, manganese uptake was reduced in areas of hypertrophy and virtually absent in areas of severe fibrosis, demonstrating a pattern of uptake similar to that of bloodpool. This suggests that MEMRI can distinguish between dysfunctional and nonviable cardiomyocytes as seen in patients with transmural acute myocardial infarction.^32^, ^61^ This ability to differentiate and track viable myocardium could play a role in the assessment of reversible causes of cardiomyopathies, which show transient myocardial dysfunction.

#### CORONAVIRUS DISEASE 2019

During the coronavirus disease 2019 (COVID‐19) pandemic, there was major concern surrounding the extent of cardiac involvement and its consequences in patients with COVID‐19. Myocardial injury was commonly seen in patients hospitalized with COVID‐19 and correlated with disease severity and poor clinical outcomes.^82^ Initial studies also reported abnormal cardiac imaging findings^83^ although many patients had concomitant cardiovascular risk factors and comorbidities, and there was an absence of appropriate comparator control subjects.

**FIGURE 9:**
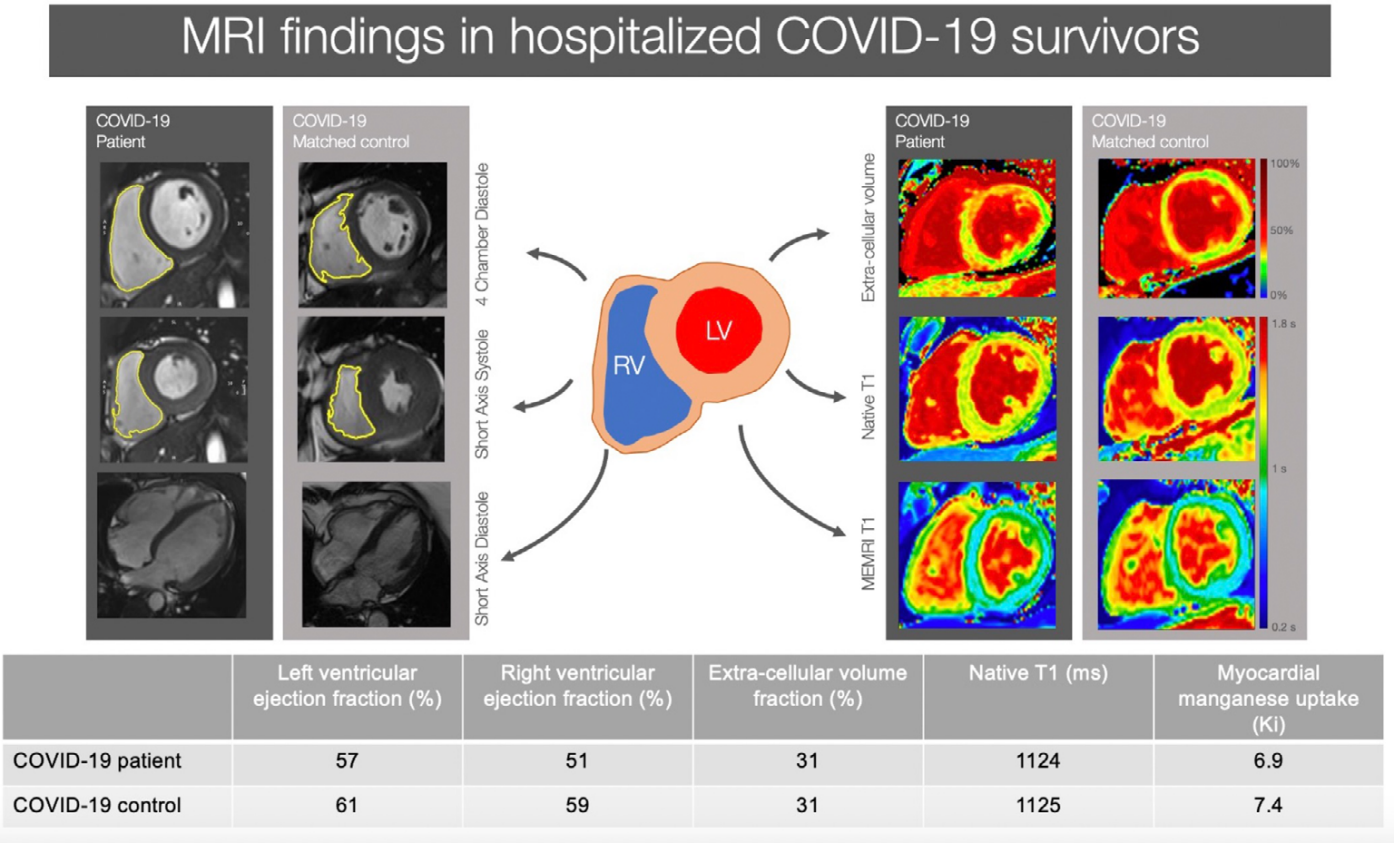
Cardiac magnetic resonance features of patients hospitalized with coronavirus disease‐19. Magnetic resonance imaging findings in patients recovering from COVID‐19 infection compared to age‐, sex‐, and co‐morbidity‐matched volunteers. MEMRI = manganese‐enhanced magnetic resonance imaging; LV = left ventricle; RV = right ventricle.

Indeed, cardiovascular risk factors and comorbidities accounted for many of the observed abnormalities, and subsequent studies demonstrated that there was no excess left ventricular dysfunction between co‐morbidity matched control subjects and those who had been hospitalized with, and recovered from, COVID‐19.^31^ However, patients did have a higher prevalence of right ventricular dysfunction, likely attributable to recent severe viral pneumonia and consequent pulmonary hypertension. The absence of persistent COVID‐19‐induced myocardial dysfunction was also confirmed MEMRI^31^ (Fig. 9). Left ventricular cardiac function and myocardial manganese uptake in patients hospitalized with COVID‐19 were similar to that of age, sex and co‐morbidity matched control subjects. This may suggest that MEMRI may therefore have the potential to detect subclinical myocardial dysfunction.

## Potential Clinical Applications and Future Directions

The potential clinical applications of MEMRI are numerous and include diagnosis, risk stratification, and management of a range of patients with cardiac disease. This technique has particular implications for diagnosis, especially for those with an uncertain or subclinical cardiomyopathy. This could include a range of cardiomyopathies including genetic causes of cardiomyopathy with variable penetrance or athlete's heart. Athlete's heart is a term referring to a constellation of electrical, functional, and structural remodeling that accompanies regular athletic training. This is an important physiological adaption, which helps the physical performance of athletes. What was initially thought to be a benign adaptation to endurance training, we now know that 20% of elite athletes demonstrate residual cardiac chamber enlargement despite detraining.^84^ This raises the question of whether athlete's heart is truly benign and how best to identify those at risk. Thus, the ability to diagnose an underlying cardiomyopathy prior to gross left ventricular dysfunction, will allow for early detection and prompt treatment initiation, which may have an impact on outcomes. However, this has yet to be established for MEMRI.

The ability to detect altered calcium handling over time and quantify cellular myocardial function directly may transform our ability to assess myocardial function, enabling early detection and prognostication. This may be an invaluable non‐invasive method of monitoring disease progression in various nonischemic cardiomyopathies. With optimization, this technique has potential to allow individualization of heart failure treatment, assessment of treatment efficacy and targeting optimal therapy to those most likely to benefit. A randomized controlled trial has confirmed that 40% of patients that have recovered from dilated cardiomyopathy (resolution of left ventricular ejection fraction) will “relapse” following discontinuation of heart failure medication.^85^ The ability to detect subclinical myocardial calcium mishandling in this population could potentially identify which individuals should continue with their heart failure treatment. Such applications are conceptual and speculative but MEMRI does now provide the opportunity to explore such approaches in future studies and potentially provide a more targeted or personalized approach to the treatment and management of our patients with cardiomyopathy.

There remains considerable interest in the development of therapies to improve the recovery of the cardiac function following myocardial infarction and coronary revascularization. MEMRI can identify viable myocardium within the peri‐infarct region and therefore presents a biomarker measure of novel treatment interventions to reduce infarct size. In the field of myocardial stem cell therapy, manganese uptake can be used as a measure of successful delivery and function of implanted stem cells as demonstrated following human amniotic mesenchymal stem cell delivery in a porcine model of acute myocardial infarction.^86^

In the past, we have been unable to quantify cellular myocardial function. With MEMRI, we are now able to assess intracellular myocardial calcium handling. The presence of late‐gadolinium enhancement has been proven to carry a poor prognosis in different cardiac conditions such as ischemic cardiomyopathy, dilated cardiomyopathy and hypertrophic cardiomyopathy.^79^, ^87^ However, to date, there has been no assessment of the prognosis of patients with dysfunctional myocardial calcium handling and it will be important to establish whether abnormal myocardial manganese uptake is associated with adverse outcomes and can provide useful prognostic information.

### Ongoing Studies

Patients with diabetes mellitus are at high risk of developing heart failure and often present with breathlessness, despite normal gross cardiac function.^88^ The use of manganese‐enhanced magnetic resonance is being assessed in the detection of preclinical cardiac dysfunction in patients with diabetes mellitus with normal left and right ventricular function. Early detection of altered calcium handling in at‐risk cardiomyopathy may enable initiation of preventative or disease‐modifying therapy earlier than previously possible, which has potential to improve long‐term clinical outcomes. A current study is investigating the role of sodium‐glucose transporter 2 (SGLT‐2) inhibitor therapy in the treatment of heart failure in patients with and without diabetes mellitus with the use of MEMRI (NCT04591639). The mechanism in which SGLT‐2 inhibitors exert beneficial outcomes is not fully understood and may involve improvements in cardiac energetics.^89^ With optimization, this technique has potential to track treatment response noninvasively. The benefits could include individualization of heart failure treatment and targeting optimal therapy to those most likely to benefit. Furthermore, this can be potentially be used to assess a wider range of cardiomyopathy phenotypes.

MEMRI has been recently assessed in patients with takotsubo syndrome (NCT04623788). It was long thought that there is complete recovery of cardiac function in patients with takotsubo syndrome. We now know that not only do patients demonstrate ongoing symptoms with abnormalities of cardiac energetics, but they are also at higher risk of morbidity and mortality, similar to those with acute myocardial infarction.^90^ Singh et al demonstrate profound abnormality in myocardial manganese uptake in the acute phase of takotsubo syndrome, which persists during convalescence, despite restoration of left ventricular function (Singh et al, unpublished data). This is the first description of abnormal myocardial calcium handling in takotsubo syndrome. Furthermore, persistent myocardial calcium mishandling may suggest patients develop a long‐term “heart failure” like syndrome, which could explain ongoing symptoms adverse long‐term outcomes.

It would be of interest to see whether MEMRI can detect myocardial calcium handling in reversible, focal cardiac pathologies and, more importantly, whether this as well as conventional measures of cardiac function recover during convalescence. An ongoing study is investigating the role of this technique in patients with acute myocarditis (NCT04623788). One in 20 patients with acute myocarditis will go on to develop heart failure^91^ and earlier detection of abnormal myocardial calcium handling may help guide earlier treatment to prevent gross left ventricular systolic dysfunction and thereby improve outcomes.

### Outside the Heart

Beyond the myocardium, manganese‐based contrast media have the potential to provide functional assessment in other organs, such as the kidneys, pancreas, and brain. Renal, pancreatic, and brain tissue have substantial manganese uptake (Fig. 2), and this could provide a noninvasive measure of cellular calcium handling prior to the onset of renal failure, pancreatic insufficiency or disease monitoring in neurodegenerative conditions.

#### KIDNEYS

L‐type calcium channels are expressed and are of significance in the renal cortical preglomerular vessels, juxtamedullary efferent arterioles, and outer medullary vasa recta. As such, manganese uptake and retention into the renal cortex via calcium channels in viable renal issue has been demonstrated and long recognized.^3^ Currently, renal function is assessed indirectly, using serum markers such as, creatinine, urea, and estimated glomerular filtration rate. However, this is not suitable to detect early stages of disease and is frequently inaccurate when compared to reference methods. This is likely related to nonfunctional factors, including unmeasured muscle mass and tubular secretion of creatinine.^92^ There is an urgent need to develop a noninvasive technique to assess renal function and viability.

Jiang et al have implemented a T1‐mapping method to obtain quantitative information both dynamically and over a range of manganese chloride concentrations in a murine model of unilateral renal artery stenosis.^93^ They detected decreased cellular viability and discerned the regional responses to renal artery stenosis. Early human clinical studies^94^ have described visual uptake of manganese by renal tissue, but dedicated renal imaging using MEMRI has yet to be investigated.

#### PANCREAS

Type 1 diabetes is characterized by autoimmune loss of pancreatic beta cell mass leading to metabolic dysregulation, requiring lifelong insulin therapy. The degree of beta‐cell dysfunction at this time often exceeds the percentage beta cell loss, suggesting additional functional impairment in insulin secretion in these patients. The beta‐cell deficit in type 1 diabetes mellitus provides a rationale for novel therapeutic strategies aimed at restoring or at least preventing further loss of beta cell mass. In the last decade, MEMRI has been used to detect declining pancreatic beta‐cell function in diabetes mellitus. Traditional gadolinium‐based contrast media are ineffective at imaging pancreatic beta cells due to their tendency to accumulate in the extracellular space. As a calcium analog, manganese is taken up into pancreatic beta cells during insulin secretion via voltage gated calcium channels, serving as an intracellular contrast agent that represents active insulin secretion.^5^

Mouse models of type 1 diabetes mellitus demonstrated a decrease in manganese uptake in T‐cell receptor transgenic nonobese diabetic mice, with normal uptake in non‐diabetic mice. This mirrored loss of beta‐cell function, which was confirmed by pancreatic insulin measurements and histology.^5^ Several other preclinical studies have shown potential for noninvasive detection of changes in functional beta‐cell mass.^95^

The proof‐of‐concept studies have demonstrated that MEMRI can distinguish between patients with type 2 diabetes mellitus and normoglycemic control subjects.^96^ An ongoing study (NCT05298735) is investigating the role of MEMRI of the pancreas in patients with type 1 and type 2 diabetes mellitus. This could provide a noninvasive measure of pancreatic beta‐cell function, which holds promise in monitoring of disease progression and the assessment of treatment response.

#### BRAIN

Lin and Koretsky were the first to demonstrate that manganese contrast can be used as a noninvasive direct measurement of neuronal function.^4^ Understanding of the biological mechanisms of manganese developed in conjunction with advances in its uses for imaging purposes, beginning in the early 1980s when its accumulation, permeability and calcium channel competition in nerve terminals was discovered. Synaptic manganese is taken up by the postsynaptic neuron through any of a number of potential calcium‐permeable channels or receptors and is repackaged for further transport propagation.

There were significant issues with manganese‐based brain imaging due to the fact that manganese cannot cross an intact blood–brain barrier. Early studies involved administering manganese chloride via a peripheral intravenous injection and reported imaging enhancement. However, this initial application of manganese‐enhanced imaging was limited by the need of a chemical or mechanical disruption in the blood–brain barrier. Systemic administration using higher doses was utilized which provided sufficient enhancement, but was complicated by toxicity and required much longer acquisition times.^4^ Subsequent studies have utilized different delivery techniques, both systemically (fractionated and continuous infusions)^97^ and locally.^98^

Animal models of neurodegenerative diseases with manganese‐enhanced imaging mainly focus on anatomy and cytoarchitecture. Following intravenous administration of manganese, contrast in the brain is achieved 24 hours later. Furthermore, reduced manganese uptake was seen in the hippocampus and the amygdala in models of Alzheimer's disease.^99^

In humans, MEMRI of the brain has been limited. Suto et al^100^ are the first to use manganese in patients with multiple sclerosis in whom there was evidence of blood–brain barrier disruption. This demonstrated enhancement of active lesions with a temporal and spatial profile distinct from that of gadolinium. Interestingly, chronic lesions did not enhance. The mechanism of manganese accumulation is not yet fully understood. Further study is needed to understand the mechanism of contrast enhancement as well as cell uptake.

### Next Steps

Since withdrawal from the European market in 2012 by the marketing‐authorization holder, no manganese contrast medium has been clinically available. The reasons for this are multifactorial but the decision was principally driven by the lack of large‐scale commercial interest, despite promising clinical pilot data. It is important to highlight that no safety concern caused its withdrawal, simply the lack of clinical demand in hepatobiliary imaging. Furthermore, early clinical studies have established that manganese is safe in various cardiac conditions.^31^, ^32^, ^33^ There are currently no available preparations of manganese‐based contrast media for widespread clinical use and has never been licensed for cardiac applications. This continues to limit the use of MEMRI in research and clinical settings. However, the formulation of manganese dipyridoxyl diphosphate for clinical use is clearly feasible, as evidenced by its current use in clinical studies (Table 1). The provision and availability of manganese‐based contrast media is likely to change with the re‐emergence of commercially available preparations anticipated in the near future.

The next step in the development of MEMRI of the heart would be to use this technique in larger clinical programs and multicenter trials. This will allow for further assessment of its use across a wider population, different T1‐mapping sequences and magnetic resonance scanners, as well as providing best evidence of effectiveness.

## Conclusion

With a large body of preclinical data and emerging clinical work in the field, the stage is now set for wider clinical translation of this exciting noninvasive imaging technique. MEMRI offers the potential to improve diagnosis in a range of conditions and to provide a noninvasive measure of myocardial calcium handling. This represents an invaluable tool for the assessment of functional recovery, accurate prediction of disease progression and monitoring of treatment response.

## Conflicts of interest

None.
